# Spinal cord stimulation patterns of care, re‐interventions, and costs for private health insurers, Australia, 2011–22: a retrospective observational study

**DOI:** 10.5694/mja2.70001

**Published:** 2025-07-14

**Authors:** Caitlin MP Jones, Christopher G Maher, Rachelle Buchbinder, Ian A Harris, Chung‐Wei Christine Lin, Christopher Hayes, Alexandra Gorelik

**Affiliations:** ^1^ The University of Sydney Institute for Musculoskeletal Health Sydney NSW; ^2^ Sydney School of Public Health the University of Sydney Sydney NSW; ^3^ Monash University School of Public Health and Preventive Medicine Melbourne VIC; ^4^ South Western Sydney Clinical School University of New South Wales Liverpool NSW; ^5^ Hunter New England Health Newcastle NSW

**Keywords:** Spinal cord diseases, Pain, Prosthesis implantation, Pain management

## Abstract

**Objectives:**

To investigate spinal cord stimulation patterns of care, the proportions of people who require unplanned surgical interventions after receiving definitive spinal cord stimulator implants, and the costs to private health insurers in Australia.

**Study design:**

Retrospective observational study; analysis of deidentified private health care insurers benefits payments data.

**Setting, participants:**

People admitted to hospital for spinal cord stimulation‐related surgical procedures, 11 January 2011 – 13 April 2022, with full or partial costs coverage by five general private health care insurers.

**Main outcome measures:**

Patterns of care; proportions of people with stimulator implants who subsequently require surgical re‐intervention, overall and within 36 months of receiving definitive implants; costs to insurer for trial, definitive implantation, and re‐interventions.

**Results:**

We analysed data for 11 451 admissions of 5839 people; mean age at first admission was 60.2 years (standard deviation, 15.4 years), 3717 people were women (63.7%). Median follow‐up time was 48 months (interquartile range [IQR], 33–72 months). Definitive stimulators were implanted in 4361 people (74.7%), of whom 3244 had previously had at least one stimulation trial (74.3%; one trial only: 2970 people); 1478 people (25.3%) had trials but never proceeded to definitive implants. Surgical re‐interventions were required by 1011 people with definitive implants (23.2%); the median time to the first re‐intervention was 16.8 months (IQR, 6.2–39.8 months). The cumulative probability of requiring surgical re‐intervention at 36 months was 0.35. The median cost to the insurer of a trial implant was $13 689, for a definitive implant (device, medical, and hospital costs for initial procedure and re‐interventions) $55 635.

**Conclusions:**

About one in four people will require surgical re‐intervention within 36 months of receiving a definitive spinal cord stimulator, and the costs for the procedure are high. Both findings are concerning given the paucity of evidence for their efficacy in treating chronic pain.

**The known:** Spinal cord stimulators are increasingly used for treating chronic pain despite the lack of evidence for their efficacy, and reports of adverse effects.

**The new:** The implanting of spinal cord stimulators is preceded by at least one trial in three of four people who receive them. About one‐third of people with the implants subsequently require a further, unplanned surgical procedure, often within three years. Definitive implants cost private health insurers more than $50 000 each.

**The implications:** Follow‐up surgical interventions are frequent in people with spinal cord stimulators, and they are also very expensive. In the absence of convincing evidence for their efficacy for reducing chronic pain, their use should be reconsidered.

Spinal cord stimulators are implantable devices for modulating nociceptive signals travelling along the spinal cord.^1^ The devices include a pulse generator, usually implanted under the skin in the buttocks, and leads implanted in the epidural space.^1^ They are promoted as treatments for certain chronic pain conditions.^2^

The use of spinal cord stimulation for pain control is not supported by high quality evidence.^3^, ^4^, ^5^, ^6^ A 2023 Cochrane review of thirteen trials found moderate certainty evidence that spinal cord stimulation probably does not provide benefits for people with back pain that outweigh its risks and costs.^5^ A 2021 Cochrane review of fifteen clinical trials found very low certainty evidence that spinal cord stimulation may not provide clinically important benefits for people with chronic pain; the reviewed studies reported adverse events in as many as 55% of participants, and 5‐year re‐intervention rates as high as 94%.^6^ An Australian study found that reported harms included infections, lead migration, increased pain, and dural punctures; 93% of harms were serious, and 83% required surgery for correction.^7^, ^8^

Spinal cord stimulation treatment usually starts with the trial implantation of temporary leads in the epidural space and an external pulse generator.^9^, ^10^, ^11^ Trial leads, substantially less expensive than definitive leads, should be used when available. If the patient reports pain relief, permanent leads and a pulse generator are implanted under the skin in a second procedure.

The costs of spinal cord stimulation comprise those for the device, programming software, surgical implantation, and follow‐up care, which often involves hospitalisations, including for planned battery changes and unplanned removal following adverse events or lack of efficacy. A United States workers’ compensation payer estimated that the medical costs over two years exceeded US$50 000 in 2007.^12^ The costs and proportions of people who require further procedures have not been examined in Australia.

Spinal cord stimulation is provided in both public and private health care in Australia, but as 90% of implants are inserted in private health care, private insurers are a good source of information about costs and re‐intervention rates.^13^ We therefore investigated spinal cord stimulation patterns of care in Australia, including the use of trial and definitive leads; the proportion of people with definitive implants who subsequently required surgical re‐interventions, the influence of sex, age, and time period of definitive implant on the risk of re‐intervention; and the costs to private health care insurers.

## Methods

For our retrospective observational study, we invited all twenty insurer members of Private Health Australia to submit records of benefit payments for spinal cord stimulation‐related services for pain treatment (excluding out‐of‐pocket costs for patients). Five Australian private health insurers (representing 76% of people with private health insurance), all general insurers (ie, not restricted or industry‐specific), provided de‐identified data from the period 11 January 2011 – 13 April 2022. Data were not available for hospital admissions in which a charge for hardware was not recorded (eg, repositioning migrated leads), and we excluded data for admissions in which neither a generator nor a lead was recorded as being used. The outcomes of interest were whether people had second interventions (binary outcome) and time to first re‐intervention.

The health funds provided costs data in various formats; one fund provided itemised costs (individual costs for trial leads, definitive leads, generators). All funds provided costs data as overall benefit payment summaries, but as they used different classification systems we used data from the Australian Therapeutic Goods Administration Prostheses List^14^ to estimate costs per treatment pathway.

### Data definitions

“Admission” refers to a hospital admission during which a billable spinal cord stimulation‐related procedure was undertaken. “First procedure” refers to the first recorded procedure, which could be a trial or a definitive implantation. A “trial” is the use of either a trial lead (wire) or a definitive lead without an implanted generator. “Definitive implantation” is the implantation of a generator. “Re‐intervention” is a surgical procedure after definitive implantation of a generator.

We assumed that re‐interventions within 36 months were unplanned, unlike those beyond 36 months, which would include battery changes, usually required every 5–10 years.^15^ We therefore report re‐interventions within 36 months separately from the primary analysis, which included all re‐interventions.

### Statistical analysis

#### Patterns of care

We summarise the characteristics of patients and general patterns of spinal cord stimulation care as means with standard deviations (SDs) or medians with interquartile ranges (IQRs) (continuous data), or as numbers and proportions (categorical data). We examined treatment pathways using sequence analysis of the sequentially ordered admissions of each person, from index admission to final follow‐up; people were identified by their unique health care fund customer identification numbers. The index admission was defined as any admission for inserting trial or definitive leads (with or without a generator). We then constructed a summary diagram of the clinical intervention paths for all patients.

#### Re‐interventions

We classified patients by the period of their first spinal cord stimulation‐related procedure, defined in consultation with clinicians to reflect changes in clinical practice and new hardware becoming available: 11 January 2011 – 31 December 2014, 1 January 2015 – 31 December 2018, or 1 January 2019 – 13 April 2022. The third group included people for whom we had less than 36 months of follow‐up data. We assessed the statistical significance of the difference between the first two periods in the proportions of people who required re‐intervention within three years of the index procedure (Pearson χ^2^ test).

A proportional hazards model was used to investigate time to re‐intervention, overall and by period of definitive implant; we provide Kaplan–Meier plots. We assessed the influence of sex, age, and period of definitive implantation on the risk of re‐intervention within 36 months of definitive implantation using proportional hazards Cox regression models; we report adjusted hazard ratios (aHRs) with 95% confidence intervals (CIs). Proportional hazard assumptions were tested using both graphical (Stata command *stphplot*) and, following the regression analysis, analytical methods (Stata command *estat phtest*); *P* was greater than 0.1 both globally and for both individual covariates, indicating that proportionality assumptions were met.

#### Costs

We report costs from the perspective of the private health insurer as nominal Australian dollars for the year in which they were incurred. The costs data are summarised as the total cost of the intervention, including the costs of individual devices, trials, definitive implantation procedure, and re‐intervention, both overall and by time of the first recorded procedure by period (2011–2015 or 2016–2019). All costs data are summarised as medians with IQRs; the statistical significance of differences between the two periods was assessed in Mann–Whitney *U* tests. We conducted sensitivity analyses in which we estimated the overall cost during three or five years of follow‐up.

All analyses were performed in Stata 17; *P* < 0.05 was deemed statistically significant.

### Ethics approval

The Sydney Local Health District Human Ethics Board approved the study (2024/ETH01237).

## Results

Insurer benefits payments data were available for 12 535 hospital admissions of 6283 unique patients who underwent spinal cord stimulation‐related procedures during 11 January 2011 – 13 April 2022 (admissions by fund: 924 [7.4%], 2270 [18.1%], 2923 [23.3%], 4545 [36.3%], 1873 [14.9%]). The median length of hospital stay was one day (IQR, 1–2 days); the mean age at first admission was 60.1 years (SD, 15.3 years; range, 14–96 years), 4034 patients were women (64.2%).

After excluding 1084 admissions (8.6%) during which neither a generator nor a lead was used, we included data for 11 451 admissions of 5839 people in our analysis; the mean age at first admission was 60.2 years (SD, 15.4 years; range, 14–96 years), and 3717 patients were women (63.7%). The median follow‐up time from the index procedure was 48 months (IQR, 33–72 months).

### Treatment pathways

#### Trials for people who never received definitive implants

A total of 1478 people (25.3%) had trials but did not have definitive generators implanted: 1193 had one trial only (80.7%), 285 had more than one trial (19.3%; median, two trials; IQR, 2–2 trials; range, 2–6 trials) (Box 1). For people who underwent only one trial, trial leads were used in 252 cases (21.1%), definitive leads in 931 (78.0%), and both lead types in ten (0.8%). The lead proportions were similar for people who underwent more than one trial (data not shown).

Box 1 Treatment pathways of members of five private health care funds admitted to hospital for spinal cord stimulation‐related procedures in Australia, 2011–22*

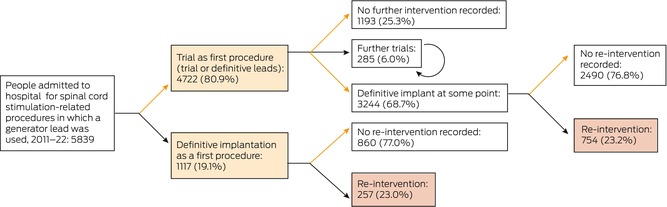

* Trial: use of definitive or trial leads without implantation of a generator. Circular arrow: multiple trials, without definitive implant.

#### Definitive implants (implantation of a generator)

Definitive generators were implanted at some time in 4361 people (74.7%), of whom 3244 had previously had at least one trial (74.3%) (Box 1): one trial for 2970 people (91.6%), two trials for 243 people (7.5%), and three or more trials for 31 people (1.0%). Of the 4361 definitive implants, 3813 generators (87.4%) were implanted during 1 January 2015 – 13 April 2022 (Box 2).

Box 2 **Re‐interventions after definitive spinal cord stimulator implant procedures for 4361 people, by period of implant procedure**

**Table:** 

Outcomes	11 Jan 2011 – 31 Dec 2014	1 Jan 2015 – 31 Dec 2018	1 Jan 2019 – 13 Apr 2022*
Definitive implants	548 (12.6%)	2227 (51.1%)	1586 (36.4%)
Re‐interventions^†^	236 (43.1%)	617 (27.7%)	158 (10.0%)
Within 36 months	157 (28.7%)	497 (22.3%)	158 (10.0%)

* Three‐year follow‐up data not available for people in this group.

† The characteristics of the 949 first re‐interventions for each person are reported in the Supporting Information, table 1.

#### Re‐interventions in people with definitive implants

Of the 4361 people with implanted generators, 3350 (76.8%) did not require re‐interventions and 1011 (23.2%) underwent at least one re‐intervention that was recorded in the dataset (Box 1, Box 2). A total of 1692 re‐interventions were undertaken (median, one per person; IQR, 1–2 per person); the median time to first re‐intervention was 16.8 months (IQR, 6.2–39.8 months).

For 811 people (18.6% of people with definitive implants, 80.2% of people who underwent re‐interventions), the re‐intervention was within 36 months of the definitive implant procedure (Box 2). The cumulative probability of re‐intervention 36 months after the definitive implant was 0.35; the probability differed by period of implant: 2011–2014, 0.46; 2015–2018, 0.38; 2019–22, 0.24 (Box 3).

Box 3 **Time to first re‐intervention after definitive implantation of spinal cord stimulators in 4361 people, overall and by time period of implant**

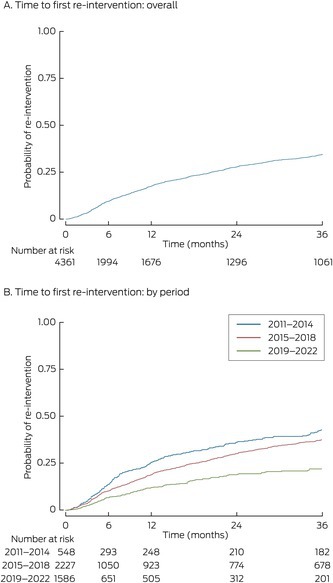

The proportion of people with index procedures during 2011–2014 who required re‐interventions within 36 months of receiving a definitive implant (157 of 548, 28.7%) was larger than for those with index procedures during 2015–2018 (497 of 2227, 22.3%; *P* = 0.002). The risk of re‐intervention during the 36 months after the index definitive implantation was similar for men and women (aHR, 0.97; 95% CI, 0.85–1.12), but declined slightly by age (per year: aHR, 0.99; 95% CI, 0.98–0.99) and was lower during 2015–2018 than during 2011–2014 (aHR, 0.69; 95% CI, 0.61–0.79).

### Costs

Only one fund provided itemised costs by device type; it contributed costs data for 2257 of 5839 people included in the analysis (38.7%). The median benefits paid for definitive leads ($8122; IQR, $7634–$8460) were higher than for trial leads ($970; IQR, $876–$1455). The median cost of a generator was $23 465 (IQR, $22 000–$24 960) (Supporting Information, table 2).

The overall median care cost for spinal cord stimulation‐related hospital admissions (device, medical, and hospital costs for initial procedure and re‐interventions) was $55 635 (IQR, $45 937–$73 023) for admissions with definitive implants and $13 689 (IQR, $10 056–$21 014) for trials. The median device costs were $11 648 (IQR, $6968–$33 151), the median admission medical costs $2857 (IQR, $1728–$4928); the device cost was higher when generators were used ($43 812; IQR, $37 296–$54 438) than when they were not ($8460; IQR, $4566–$13 362), as were medical costs ($6318; IQR, $3405–$10 779 *v* $3256; IQR, $2034–$5254) (Box 4).

Box 4 **Costs per patient for trial and definitive implantations of spinal cord stimulators: device, medical, and hospital costs (initial procedure and re‐interventions), nominal Australian dollars**

**Table:** 

	Definitive implants (generator used)		Trial only (no generator used)	
Cost type	Median (IQR)	Range	Median (IQR)	Range
Number of episodes	4361		1478	
Device	43 812 (37 296–54 438)	963–283 623	8460 (4566–13 362)	485–74 078
Medical	6318 (3405–10 779)	70–227 372	3256 (2034–5254)	64–58 358
Hospital*	18 191 (13 684–40 471)	1219‐267618	7994 (6710–13 689)	671–56 905
Medical and device	51 461 (42 658–64 342)	514–356 269	11 857 (8914–18 203)	1129–90 617
Medical, device, and hospital	55 635 (45 937–73 023)	513–528 256	13 689 (10 056–21 014)	1137–110 920

IQR = interquartile range.

* Provided only by some funds; for definitive implants, data were available for 1099 people; for trial procedures, 347 people.

The median device cost was similar during 2011–2015 and 2016–2020, but the difference was statistically significant because of outliers (very high costs) during 2016–2020 associated with interventions such as the implanting of multiple new generators over several years (Supporting Information, table 3). Sensitivity analyses that limited costs to those incurred within three (Supporting Information, table 4) or five years of the index implantation procedure (Supporting Information, table 5) yielded similar results.

## Discussion

In our analysis of Australian private health insurance data for 11 451 hospital admissions of 5839 people who underwent spinal stimulation‐related procedures during January 2011 – April 2022, 4361 people ultimately received definitive implants, in 2970 cases (68%) preceded by single stimulator trials, but variation in the pattern of care was substantial. Re‐intervention within three years of receiving definitive implants was required by 28.7% of people who received them during 2011–2014, 22.3% during 2015–2018, and 10.0% during 2019–22; the likelihood of re‐intervention declined with age. The median cost to one insurer for an episode of care (including all related re‐interventions over median follow‐up time of 48 months, but excluding costs directly borne by the patient) was $13 689 for trials and $55 635 for definitive implants. The variation in costs was marked, the range of total costs spanning more than $500 000 for definitive implants and $100 000 for trials.

We found that the overall probability of re‐intervention within 36 months of receiving a definitive implant was 0.35, which is similar to overseas findings. Three small single centre studies in the United States (100–291 patients) reported re‐intervention rates of 31% to 67%; the median time to first re‐intervention ranged between 16 and 43 months.^16^, ^17^, ^18^

Definitive leads cost substantially more than trial leads, but we found that definitive leads were often used in trials. As more than 30% of trials were not followed by definitive implant procedures or were followed by further trials, substantial costs could be saved by using trial rather than definitive leads for trial procedures. Further, the proportions of people who required re‐interventions after definitive implant surgery was similar for those who had first undergone trials and those who had not (about 23%).

Our findings supply information to consider when making decisions about funding these devices. Spinal cord stimulation provided in Australian private health care is associated with high costs and high re‐intervention rates. Our findings also need to be considered alongside the findings of two Cochrane reviews that spinal cord stimulation may provide limited to no benefit for people with chronic pain, including back pain, neck pain, nerve pain, and complex regional pain syndrome.^5^, ^6^ On the other hand, spinal cord stimulation could cause harm.^7^

Spinal cord stimulation should be offered as a treatment option in Australia only in the context of a randomised controlled trial and a parallel economic evaluation. Trial or real world clinical outcomes data could be reported to the electronic Persistent Pain Outcomes Collaboration (ePPOC), which collects and reports pain outcomes from pain management services in Australia and New Zealand.^19^ This reporting would facilitate comparisons of outcomes with a package of care that includes spinal cord stimulation with those with a package of care that does not.

### Limitations

We analysed a dataset covering more than ten years to estimate both the risk to patients (re‐interventions) and costs to health insurers. The authors have no financial connections with device manufacturers or private health insurers. However, as we did not have information about reasons for re‐interventions, we cannot comment on their appropriateness. Planned or predictable re‐interventions, such as battery changes or pulse generator upgrades, would not be expected during the median follow‐up time of 48 months (IQR, 33–72 months). The third time period in our study (1 January 2019 – 13 April 2022) included the coronavirus disease 2019 (COVID‐19) pandemic, which may have affected our findings because of the cancellation of non‐urgent surgery.

We have probably underestimated the number of re‐interventions, as we excluded admissions in which neither a generator nor a lead was used (1084 admissions involving other devices, such as lead extensions, patient programmers, intraoperative accessories). We excluded these admissions because we could not be certain that these interventions were linked with surgical procedures and could therefore be classified as re‐interventions. This means, however, that we excluded procedures for repositioning migrated leads, one of the most frequently reported adverse events.^7^

Further, we have probably underestimated the number of re‐interventions because we could not track people across different health insurers; re‐interventions for people who changed insurer or left private health insurance altogether will have been missed. We did not have information about treatment effectiveness; for instance, people may have found devices ineffective but elected to leave them in place. We could assess costs data but not cost‐effectiveness. We underestimated total costs, as we did not include out‐of‐pocket costs for patients or costs to anyone other than the private health insurer. We did not know the level of insurance coverage for each patient. We did not have information about relevant presentations to emergency departments or other related health care use and its costs. As our costs data were provided by only five health care insurers, our findings may not be generalisable to all Australian health insurers, or to insurers in other countries. Costs by device type were based on information from one insurer and may not be representative of all providers. We reported costs in nominal dollars without adjustment for inflation. Finally, data on the characteristics of the patients was restricted to age and sex.

### Conclusion

In our analysis of paid benefits data for 2011–22 provided by five Australian health care insurers, we found that 74.3% of definitive spinal cord stimulator implantations were preceded by at least one trial; some people had undergone multiple trials, and 25.3% of people who underwent trials never received definitive implants. Of those who received definitive implants, 23.2% subsequently required re‐interventions; the median time to first re‐intervention was 16.8 months. The median cost to the insurer of a trial implant was $13 689 per person, and for a definitive (device, medical, and hospital costs for initial procedure and re‐interventions) it was $55 635. Given the lack of supportive data for their efficacy, and a concerning harms profile, use of spinal cord stimulators for pain control should be reconsidered.

## Open access

Open access publishing facilitated by the University of Sydney, as part of the Wiley – the University of Sydney agreement via the Council of Australian University Librarians.

## Competing interests

No relevant disclosures.

## Data sharing

Individual patient data will not be shared because of privacy and confidentiality concerns.

## Supporting information


Supplementary results
