# Supplementary material for: Spinal cord stimulation patterns of care, re‐interventions, and costs for private health insurers, Australia, 2011–22: a retrospective observational study
Source: Med J Aust. 2025 Jul 14;223(5):243–7. doi: 10.5694/mja2.70001 (PMC12399048; doi:10.5694/mja2.70001)
Supplement: Supplementary file 1 — Supplementary results [file MJA2-223-243-s001.pdf]

The Medical Journal of Australia

Supporting Information

Supplementary results

This appendix was part of the submitted manuscript and has been peer reviewed.
It is posted as supplied by the authors.

Appendix to: Jones CMP, Maher CG, Buchbinder R, et al. Spinal cord stimulation patterns of care, re-interventions, and costs for private health insurers, Australia, 2011–22: a retrospective observational study. *Med J Aust* 2025; doi: 10.5694/mja2.00000.

Supplementary results

Table 1. Characteristics of 949 first re-interventions [This table, and the included data, are not mentioned in the text.]

Implanted components	Number	Time to first re-intervention (months), median (IQR)
Definitive lead	548 (54.2%)	11.3 (4.6–28.4)
Another generator	217 (21.5%)	30.4 (15.6–57.1)
Definitive lead and generator	174 (17.2%)	22.6 (7.1–45.6)
Trial lead, definitive lead, and generator	10 (1.0%)	22.4 (12.7–43.7)

IQR = interquartile range.

Table 2. Costs per device, dollars (data from one private insurer)

Item type	Median (IQR)	Mean (SD)	Range
Trial lead	970 (876–1455)	1087 (516)	438–2910
Definitive lead	8122 (7634-8460)	9163 (3532)	485-31,696
Generator	23,465 (22,000-24,960)	23,889 (4092)	4704-91,200
Other	2352 (384–3356)	2317 (2084)	152-13,444

IQR = interquartile range, SD = standard deviation.

Table 3. Overall costs (dollars), by year of index admission

A. Patients with generators (4361 people)

	2011-2015: 1164 people			2016-2019: 3197 people		
Cost type	Median (IQR)	Mean (SD)	Range	Median (IQR)	Mean (SD)	Range
Medical cost	7216 (3548–11,958)	10,348 (12,749)	232–227,372	6,052 (3337–10,488)	8045 (8040)	70–180,134
Device	43,845 (35,723–63,607)	53,800 (27,492)	20,650–247,576	43,789 (37,672 – 51,608)	47,483 (18,839)	963–283,623
Medical & Device	52,818 (41,791–74,980)	63,886 (35,713)	2116–356,269	50,962.7 (42,917–61,664)	55,498 (23,015)	514–316,194

B. No generator used (1478 people)

	2011-2015: 386 people			2016-2019: 1092 people		
Cost type	Median (IQR)	Mean (SD)	Range	Median (IQR)	Mean (SD)	Range
Medical cost	3609 (2036–5995)	5535 (6865)	102–58,358	3100 (2022–5137)	4219 (4254)	64–51,818
Device	8460 (2326–11,984)	8876 (8426)	485–50,672	8460 (7815–14,152)	10,417.3 (7,311.3)	653.0–74,078
Medical & Device	11,424 (5755–18,266)	14,411 (12,775)	1,129–81,991	11,971 (9399–18,187)	14,648 (9278)	1137–90,617

IQR = interquartile range, SD = standard deviation.

Table 4. Overall costs (dollars), by year of index admission (restricted to three years' follow-up)

A. Patients with generators: 4032 people

	2011-2015: 928 people			2016-2019: 3104 people		
Cost type	Median (IQR)	Mean (SD)	Range	Median (IQR)	Mean (SD)	Range
Medical cost	6939 (3274–10,947)	8969 (9121)	232–119,849	5999 (3325–10,423)	7908 (7887)	70–180,134
Device	43,553 (37,559–50,568)	46,427 (26,380)	963–182,714	40,938 (35,114–51,796)	46,630 (29,023)	20,650–195,923
Medical & Device	49,700 (40,572–62,084)	55,348 (24,807)	1– 256,290	50,594 (42.731–60,649)	54,260 (20,483)	1– 246,741

B. No generator used: 1462 people

	2011-2015: 376 people			2016-2019: 1086 people		
Cost type	Median (IQR)	Mean (SD)	Range	Median (IQR)	Mean (SD)	Range
Medical cost	3543 (2032–5828)	5417 (6792)	102–58,358	3107 (2047–5130)	4230 (4256)	64–51,818
Device	8460 (2326–11,648)	8508 (8064)	485-50,672	8460 (7815–14,152)	10,404 (7296)	653–74,078
Medical & Device	10,969 (5669–16,990)	13,911 (12,406)	1129–81,991	11,953 (9394–18,150)	14,591 (9266)	1137–90,617

IQR = interquartile range, SD = standard deviation.

Table 5. Overall costs (dollars), by year of index admission (restricted to five years' follow-up)

A. Patients with generators: 4247 people

	2011-2015: 1052 people			2016-2019: 3195 people		
Cost type	Median (IQR)	Mean (SD)	Range	Median (IQR)	Mean (SD)	Range
Medical cost	7017 (3437–11,358)	9760 (12,109)	232–227,372	6079 (3337–10,489)	8049 (8042)	70–180,134
Device	42,002 (35,532–57,352)	49,997 (22,318)	20,650–195,923	43,817 (37,726–51,617)	47,535) (18,778)	963–283,623
Medical & Device	51,314 (41,337–69,516)	59,482 (29,974)	232–356,269	50,963 (42,948–61,660)	55,507 (23,021)	1–316,194

B. No generator used: 1472 people

	2011-2015: 385 people			2016-2019: 1091 people		
Cost type	Median (IQR)	Mean (SD)	Range	Median (IQR)	Mean (SD)	Range
Medical cost	3585 (2035–5954)	5484 (6811)	102–58,358	3121 (2050–5146)	4244 (4256)	64–51,818
Device	8460 (2326–11,684)	8660 (8190)	485–50,672	8460 (7815–14,152)	10,436 (7296)	653–74,078
Medical & Device	11,255 (5696–17,920)	14,130 (12,570)	1129–81,991	11,971 (9404–18,172)	14,637 (9275)	1137–90,617

IQR = interquartile range, SD = standard deviation.
